# Involving patients in setting priorities for healthcare improvement: a cluster randomized trial

**DOI:** 10.1186/1748-5908-9-24

**Published:** 2014-02-20

**Authors:** Antoine Boivin, Pascale Lehoux, Réal Lacombe, Jako Burgers, Richard Grol

**Affiliations:** 1 Charles-Lemoyne Research Center, Department of Family and Emergency Medicine, Université de Sherbrooke, Sherbrooke, Canada; 2 Scientific Institute for Quality of Healthcare, Radboud University Nijmegen, Nijmegen, the Netherlands; 3 Agence de la santé et des services sociaux de l’Abitibi-Témiscamingue, Quebec, Canada; 4 Department of Health Administration, IRSPUM, Université de Montréal, Montréal, Canada; 5 Dutch College of General Practitioners, Utrecht, the Netherlands

**Keywords:** Patient participation, Quality improvement, Health policy, Primary care, Chronic diseases, Randomized trial

## Abstract

**Background:**

Patients are increasingly seen as active partners in healthcare. While patient involvement in individual clinical decisions has been extensively studied, no trial has assessed how patients can effectively be involved in collective healthcare decisions affecting the population. The goal of this study was to test the impact of involving patients in setting healthcare improvement priorities for chronic care at the community level.

**Methods:**

Design: Cluster randomized controlled trial. Local communities were randomized in intervention (priority setting with patient involvement) and control sites (no patient involvement). Setting: Communities in a canadian region were required to set priorities for improving chronic disease management in primary care, from a list of 37 validated quality indicators. Intervention: Patients were consulted in writing, before participating in face-to-face deliberation with professionals. Control: Professionals established priorities among themselves, without patient involvement. Participants: A total of 172 individuals from six communities participated in the study, including 83 chronic disease patients, and 89 health professionals. Outcomes: The primary outcome was the level of agreement between patients’ and professionals’ priorities. Secondary outcomes included professionals’ intention to use the selected quality indicators, and the costs of patient involvement.

**Results:**

Priorities established with patients were more aligned with core generic components of the Medical Home and Chronic Care Model, including: access to primary care, self-care support, patient participation in clinical decisions, and partnership with community organizations (p < 0.01). Priorities established by professionals alone placed more emphasis on the technical quality of single disease management. The involvement intervention fostered mutual influence between patients and professionals, which resulted in a 41% increase in agreement on common priorities (95%CI: +12% to +58%, p < 0.01). Professionals’ intention to use the selected quality indicators was similar in intervention and control sites. Patient involvement increased the costs of the prioritization process by 17%, and required 10% more time to reach consensus on common priorities.

**Conclusions:**

Patient involvement can change priorities driving healthcare improvement at the population level. Future research should test the generalizability of these findings to other contexts, and assess its impact on patient care.

**Trial registration:**

The Netherlands National Trial Register #NTR2496.

## Background

Patients are increasingly seen as active partners in healthcare. The rise of chronic diseases highlights the importance of productive interactions between patients and health professionals [1,2]. Internationally, a number of healthcare organizations are involving patients in health services delivery and policy decisions, including: guideline and quality indicator development, program development and evaluation, quality improvement, and funding priorities [3-8]. This growth in patient involvement includes large national organizations (*e.g.*, Patient Centered Outcomes Research Institute, and the National Institute for Clinical Excellence), as well as local healthcare organizations (*e.g.*, Primary Care Trusts, and Patient-Centered Medical Homes) [9-11].

While patient involvement is widely advocated, its actual impact on healthcare delivery and policy decisions is largely unknown. In the past decades, over 200 trial have assessed the impact of involving patients in individual clinical decisions (*e.g.*, patient decision aids, self-management support, and health education) [12-18]. In contrast, no trial assessed the impact of patient involvement on collective healthcare decisions affecting the population [19-26]. As a result, the latest Cochrane review on this topic concluded that there is ‘a huge gap in the evidence from comparative studies about desirable and adverse effects of [patient] involvement in healthcare decisions at the population level’ [19].

Deliberation theory posits that effective patient involvement could foster mutual influence and increased agreement between patients and professionals, resulting in collective decisions about healthcare services and policies that are more acceptable by those affected [24,27,28]. A number of case reports and observational studies have tested this hypothesis with conflicting results [19,21-24,29,30]. Several challenges have been identified to support patient involvement at the population level. First, lack of understanding of scientific literature or resource implications could lead to unrealistic decisions [21]. Unbalanced recruitment may underrepresent the views of vulnerable patients with complex conditions or from disadvantaged socio-economic groups [31]. Finally, a number of critics doubt that patients can actually influence professionals’ decisions and have an impact on collective healthcare choices.

This article reports on results of the first trial of patient involvement in collective healthcare decisions affecting the population. Our primary objective was to assess the impact of patient involvement on healthcare improvement priorities at the community level. We hypothesized that patient involvement would result in mutual influence between patients and professionals, and increase agreement on common improvement priorities.

## Methods

### Study setting

This study was implemented in a real-world priority-setting exercise organized by the Regional Health Authority of Abitibi-Témiscamingue (Québec), Canada. The region has a population of 145,000 people, and is divided into six local communitites. Local Health and Social Services Centers are responsible for primary healthcare delivery in each of these communitites through direct service provision and contract with primary care providers [32]. In 2010–2011, the Regional Health Authority required its six local Health and Social Services Centers to set priorities for improving chronic disease prevention and management in primary care. Each Health and Social Services Center could choose its own improvement priorities from a list of 37 validated quality indicators. Selected priorities were incorporated in their financial accountability contracts with the Regional Health Authority.

### Design

**Figure 1:**
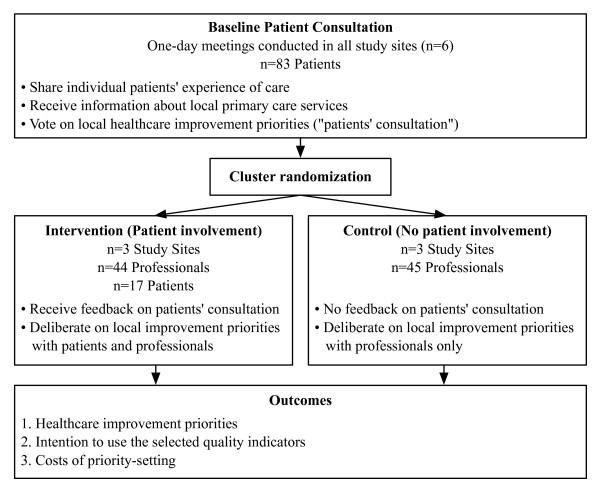
**Trial overview.** Patients and professionals were recruited from 6 eligible Health and Social Services Centers. At baseline, patients from all sites were consulted by vote on their baseline priorities. Health and Social Services Centers were then randomized in intervention (priority-setting with patient involvement) and control sites (priority-setting without patient involvement).

We conducted a cluster-randomized controlled trial comparing healthcare improvement priority-setting with and without patient involvement (Figure 1). At baseline, we collected patients’ priorities from all participating sites. Health and Social Services Centers were then randomized in intervention sites (priority-setting with patient involvement) and control sites (priority-setting by professionals alone, without patient involvement).

Our detailed study protocol has been published elsewhere [33]. We pilot-tested our intervention with 27 participants (15 patients and 12 professionals) in a pilot site outside of the study region, and tested our research instruments with an additional 21 participants (11 patients and 10 professionals) [33]. The study was conducted between January 2010 and May 2011 and was approved by the Université du Québec en Abitibi-Témiscamingue ethics committee.

### Quality indicators used as healthcare improvement priorities

In preparation for the trial, we conducted a systematic review of validated quality indicators for chronic disease prevention and management in primary care (including diabetes, chronic obstructive pulmonary disease, and cardiovascular disease) [33]. A total of 1,489 individual quality indicators were identified, 801 of which met our inclusion criteria. A panel of five experts (two physicians, two managers, and one information specialist) independently rated each indicator based on its measurability with existing information systems, and applicability to the canadian primary care context. The comprehensiveness of this preliminary indicator set was tested with a group of nine patients and 11 professionals to ensure that it included important dimensions of quality. Two indicators were added in response to patients and professionals’ comments. The final list of indicators included 37 quality indicators grouped into five domains: access, continuity and integration of care, technical quality of disease prevention and management, interpersonal relationships, and outcomes. Details of the quality indicators used in the trial are available at: http://www.implementationscience.com/content/6/1/45/additional[33].

### Recruitment

We sought to recruit the whole population of available study sites in the region (target: six communities). Within each study site, we created a recruitment team composed of the medical director, the local chief executive officer, a manager in charge of chronic disease services, and a patient sitting on the Health and Social Services Center’s user committee. Recruitment teams were responsible for identifying a diversified pool of potential participants through open advertizing, healthcare and patient organizations, and snowballing technique [34].

Selection of participants was conducted by a blinded research assistant, before randomization. The research assistant contacted potential participating patients, collected socio-demographic characteristics, confirmed eligibility criteria, and selected patients using stratified random selection to ensure a balanced representation of age, gender, socio-economic condition, and health status.We sought to recruit patients targeted by chronic disease prevention and management services, including: healthy adults without chronic disease; patients with uncomplicated chronic disease (diabetes, chronic obstructive pulmonary disease, or cardiovascular disease); and patients with complex chronic conditions hospitalized in the previous year. Patients who trained or worked as a health professional were excluded. Professionals were selected to include a balanced group of clinicians and managers involved in chronic disease prevention and management, including: primary care physicians, allied health professionals directly involved in patient care, managers responsible for chronic disease services and existing information systems, and the chief executive officer of each Health and Social Services Center.

### Patients consultation meetings

A patient consultation meeting was conducted in all sites before randomization (Figure 1). This one-day patient consultation meeting (target = 15 patients/site) was facilitated by an expert moderator. Patients recounted their personal experiences of care in relation with chronic disease prevention and management, received information about primary care services from their community, and were consulted individually by vote to select five quality indicators that they believed should be prioritized for healthcare improvement in their community (‘baseline patient consultation’).

This patient consultation meeting had two functions. First, it was used for outcome assessment in intervention and control sites, by providing baseline data on patients’ priorities for healthcare improvement. Second, the patient consultation meeting was a component of the involvement intervention aimed at preparing patients who would later participate in deliberation meetings with professionals.

### Randomization

Blocked randomization [35] of study sites was performed after participants’ recruitment, and after completion of the patients’ baseline consultation meetings. The random allocation sequence was generated by an independent expert through a randomization software [36]. Participants were thereafter unblinded to their assignment. Participants were required not to disclose any information from meetings until completion of the trial, which we verified at the end of the study. Study sites were more than 100 km apart from one another, and we found no evidence of contamination between intervention and control sites.

### Intervention

In intervention sites, Health and Social Services Centers established their improvement priorities with patient involvement. Our patient involvement intervention combined public consultation and participation methods [37], as follows: 1) professionals received feedback on priorities collected from all patients in the baseline consultation meeting; and 2) professionals and patients participated in a two-day deliberation meeting to agree on healthcare improvement priorities for their community, using nominal group technique [38]. Patients who participated in the baseline consultation meeting were invited to participate in the deliberation meeting (target = five patients/site) and were selected by a research assistant through stratified random sampling to include a balanced representation of age, gender, health status, and socio-economic conditions. Each Health and Social Services Center was allowed to select its own priorities. An expert moderator facilitated all meetings with two co-moderators.

### Control

Health and Social Services Centers in control sites established their improvement priorities with professionals only, without patient involvement. Professionals in control sites did not receive feedback on patients’ baseline priorities. Professionals deliberated among themselves to agree on healthcare improvement priorities in a two-day deliberation meeting, using similar nominal group techniques and moderators as in other sites.

### Data collection and analysis

The primary study outcome was the level of agreement between patients’ and professionals’ priorities. Secondary outcomes included changes in priorities, professionals’ intentions to use quality indicators for healthcare improvement, and the costs of patient involvement.

At baseline and at the end of the trial, each participant was asked to identify five healthcare improvement priorities from the list of 37 quality indicators. Priorities were ranked based on the proportion of participants who selected them. Agreement between patients’ and professionals’ priorities was calculated using the Spearman correlation coefficient (0 = no agreement; 1 = perfect agreement). Statistical differences in correlation coefficients between intervention and control sites were tested using Fisher r-to-z transformation [39], and we used multi-level analysis to account for the clustering of priority scores at the community level [40]. Differences in improvement priorities were calculated from the proportion of participants who selected each indicator, and tested using Generalized Estimating Equation [41]. All differences were assumed to be significant at p <0.05 (two-tailed test).

Professionals’ intention to use the selected priorities for healthcare improvement were collected at the end of the trial from an 11-item questionnaire measuring their perception of the credibility, feasibility, and importance of the selected quality indicators, as well as their intention to use them for healthcare improvement. Each item was scored on a seven-point Likert scale. Mean score differences between intervention and control sites were tested using multi-level analysis. We calculated the marginal cost of patient involvement (patient recruitment, training, and financial compensation of $100/day) in relation with total project cost (health professionals’ salary, quality indicator menu development, moderation of meetings, and project coordination). All costs were calculated from the sponsoring organization’s perspective and assumed an average of 15 patients per training meeting and five patients per deliberation meeting. All statistical analyses were conducted with SAS 9.

## Results

### Participants

A total of 172 individuals from the six eligible sites participated in the study. Characteristics of study sites and individual participants were similar in intervention and control groups (Table 1). Professionals (n = 89) included managers (35%), physicians (13%), nurses (24%), and allied health professionals (28%). Patients (n = 83) included adults with different health status: 81% of patients had at least one chronic condition, 37% had three chronic conditions or more, and 24% had been hospitalized at least once in the past year. Patients also included individuals from diverse socio-economic status: 15% had a family income of less than $20,000 and 58% had a primary or high-school level education.

**Table 1:** Characteristics of study sites and individual participants

**Sites' characteristics**		**Intervention**		**Control**	
		**(n = 3)**		**(n = 3)**	
	Average population size	25.002		23.610	
	Family physician/population ratio	1/771		1/789	
	% population 65 y.o and above	15.6%		13.4%	
	% population with low income	12.3%		11.3%	
	% population with diabetes	6.5%		6.5%	
**Professionals' characteristics**		**Intervention**		**Control**	
		**(n = 44)**		**(n = 45)**	
		**n**	**(%)**	**n**	**(%)**
Age					
	20 to 44 years	21	(47.7)	19	(48.7)
	45 to 64 years	20	(45.5)	20	(51.3)
	65 years or more	3	(6.8)	0	(0.0)
Gender					
	Male	9	(20.5)	10	(23.3)
	Female	35	(79.5)	33	(76.7)
Type of work					
	Physician	6	(13.6)	6	(13.3)
	Nurse	11	(25.0)	10	(22.2)
	Allied health professional	11	(25.0)	14	(31.1)
	Manager	16	(36.4)	15	(33.3)
**Patients’ characteristics**		**Baseline consultation**		**Deliberation**	
		**(n = 83)**		**(n = 17)**	
		**n**	**(%)**	**n**	**(%)**
Age					
	20 to 44 years	12	(14.6)	3	(18.8)
	45 to 64 years	44	(53.7)	9	(56.3)
	65 years or more	26	(31.7)	4	(25.0)
Gender					
	Male	36	(43.4)	10	(58.8)
	Female	47	(56.6)	7	(41.2)
Family income					
	Less than 20 000 $	16	(20.0)	3	(17.6)
	From 20 000 $ up to 39 999 $	29	(36.3)	8	(47.1)
	From 40 000 $ up to 59 999 $	13	(16.3)	4	(23.5)
	60 000 $ or more	22	(27.5)	2	(11.8)
Education level					
	Primary school	12	(14.8)	0	(0.0)
	High school	35	(43.2)	7	(41.2)
	College or University	34	(42.0)	10	(58.8)
Specific chronic conditions					
	Arthritis	13	(15.7)	3	(17.6)
	Cardiovascular disease	19	(22.9)	3	(17.6)
	Chronic pain	11	(13.3)	3	(17.6)
	Diabetes	30	(36.1)	2	(11.8)
	Dyslipidemia	22	(26.5)	5	(29.4)
	Hypertension	30	(36.1)	6	(35.3)
	Mood disorder	9	(10.8)	1	(5.9)
	Pulmonary disease	17	(20.5)	1	(5.9)
	Other	24	(28.9)	3	(17.6)
Number of chronic conditions					
	0	16	(19.3)	5	(29.4)
	1	21	(25.3)	5	(29.4)
	2 or more	46	(55.4)	7	(41.2)
Hospitalisations in the past 12 months					
	0	62	(76.5)	13	(76.5)
	1	10	(12.3)	2	(11.8)
	2 or more	9	(11.1)	2	(11.8)

All patients (n = 83) participated in the baseline consultation, and five to six patients per site (n = 17) also participated in the deliberation meetings with intervention site professionals. All patients completed the study and one professional did not.

### Healthcare improvement priorities

**Figure 2:**
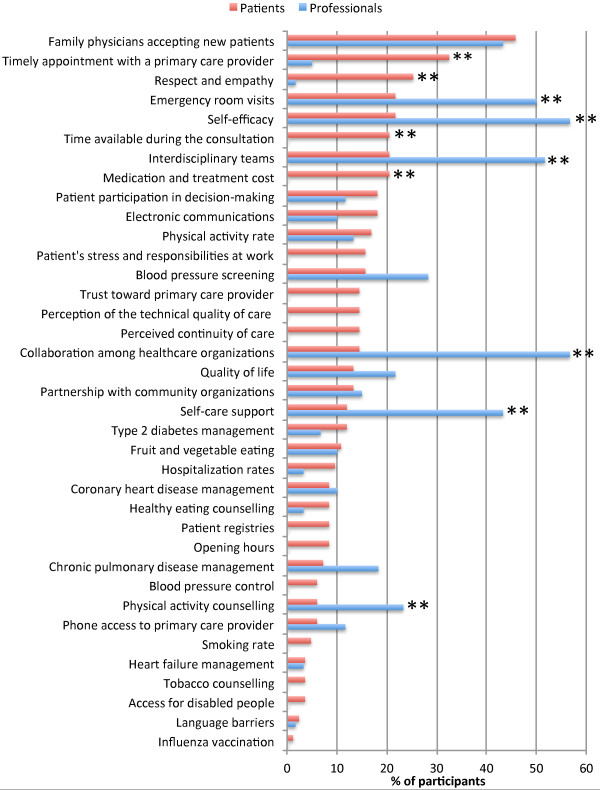
**Baseline improvement priorities for patients and professionals.** Baseline improvement priorities of patients (red) and professionals (blue). **p < 0.01.

Patients’ baseline priorities were different from those of professionals (Figure 2). Patients placed more importance than professionals on access to primary care, respect and empathy, time available in the consultation, and treatment costs (p < 0.01).

**Figure 3:**
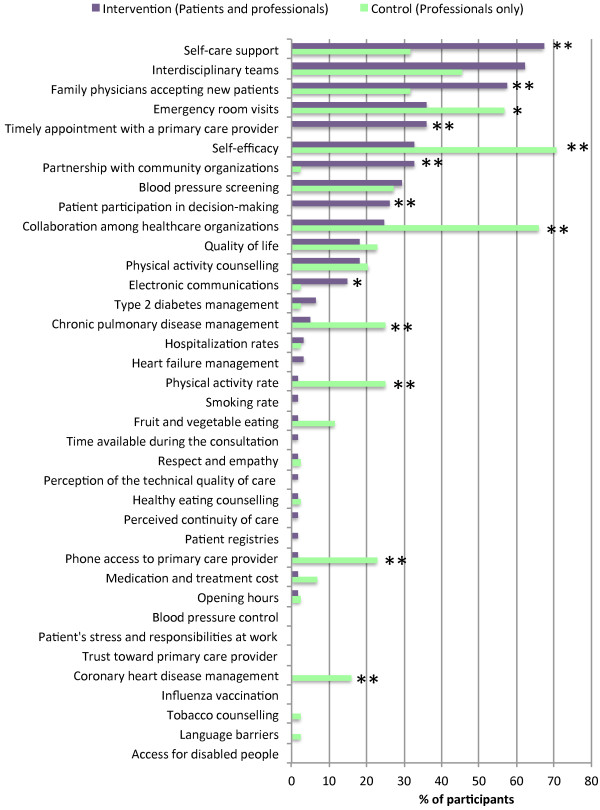
**Final improvement priorities in intervention and control sites.** Final healthcare improvement priorities in intervention sites (purple) and control sites (green). *p < 0,05 **p < 0,01.

At the end of the trial, intervention sites’ priorities identified by patients with professionals were significantly different from control sites’ priorities identified by professionals alone (Figure 3). Priorities established with patients placed more importance on generic aspects of quality, including access to primary care, self-care support, patient participation in clinical decision-making, and partnership with community organizations (p < 0.01). Priorities established by professionals alone placed more emphasis on reducing emergency room visits, collaboration among healthcare organizations, and the technical quality of single disease management (p < 0.01).

Differences in healthcare improvement priorities were explained by a process of mutual influence between patients and professionals. Intervention sites selected priorities that both patients and professionals could agreed on. Accordingly, quality indicators that were initially supported by both professionals and patients (*e.g.*, family physicians accepting new patients, patient participation in clinical decision-making, and partnership with community organizations) were selected more often in intervention sites. Initial disagreement between professionals and patients resulted in changes of opinions on both sides. For example, intervention sites’ professionals moved toward priorities that were initially favored by patients (*e.g.*, timely appointment with a primary care provider) and away from indicators that received little patient support (*e.g.*, collaboration among healthcare organizations). Patients’ priorities also moved toward indicators favored by professionals (*e.g.*, self-care support) and away from priorities that received little professional support (*e.g.*, respect and empathy). As a result of this process of mutual influence, agreement between patients and professionals increased by 41% favouring intervention sites (95%CI: +12% to +58%; p < 0 .01) (Table 2).

**Table 2:** Agreement between patients and professionals’ priorities

	**Agreement at baseline**	**Agreement at the end of the trial**	**Change in agreement during the trial**
	r	r	% change (95%CI)
Intervention	0.27	0.69	+42% (+13%, +58%)
Control	0.18	0.19	+1% (−25%, +27%)
Difference between intervention and control	+9%	+ 50%	+41% (+12%, +58%)
p value	0.62	<0.001	<0.01

Agreement between patients and professionals’ priorities at baseline and at the end of the trial (r = 0: no agreement, r = 1: perfect agreement). P value is reported for the difference between intervention and control sites.

Priorities at control sites reflected those selected by professionals at baseline, and remained at odd with patients’ priorities. For example, none of the control sites’ professionals prioritized timely appointments with a primary care provider, although this was the second most important priority identified by patients.

### Intention to use

Professionals’ intention to use selected indicators for healthcare improvement scored high in both intervention and control groups (Additional file 1). There was no difference in professionals’ perceptions of the credibility, importance, and feasibility to use selected indicators as local improvement targets.

### Costs

The average cost of public involvement was $9,427 per site. Patient involvement increased the cost of the prioritization process by 17% compared with priority-setting by professionals alone. Most of patient involvement costs were incurred by compensation of participants’ time, meal, and travel expenses (34%), coordination of patient recruitment (29%), and hiring of a professional facilitator (15%). Time to reach agreement on common priorities was, on average, 10% longer in intervention sites.

## Discussion

To the best of our knowledge, this is the first trial of patient involvement in collective healthcare improvement decisions at the population level [19,20,22,23,25]. This study shows that patient involvement can change priorities driving healthcare improvement at the community level. The involvement intervention fostered mutual influence and increased agreement between patients and professionals. We found no evidence that patient involvement adversely affected professionals’ intention to use the selected quality indicators for improvement. However, patient involvement required more time and dedicated resources than priority-setting by professionals alone.

The observed impact of patient involvement on priority-setting is clinically significant. Patient involvement shifted healthcare improvement priorities toward core generic components of the Patient-Centered Medical Home and Chronic Care Model, including timely access to a regular primary care provider, self-care support, patient participation in clinical decision-making, and partnership with community organizations [42,43]. Patients’ ability to move priorities toward access to primary care is meaningful from a North American perspective, as recent international studies rank Canada and the US last among developed countries on this quality dimension, a problem with significant documented impacts on population health [44,45].

### Policy implications

This study is important as it is the first trial to document the impact of patient involvement on strategic healthcare decisions affecting the population [19,21-23]. Such experimental study is unique and makes a significant contribution to the field. While observational studies demonstrated that deliberation influences patients’ opinions, this is the first trial evidence showing that patients can influence professionals and have an actual impact on collective decision-making [29]. Our ability to involve vulnerable patients from disadvantaged socio-economic groups in a real-world priority-setting exercise is also important, and supports the feasibility of effectively involving a broad range of stakeholders in complex policy decisions [31].

These findings have practical implications for the development of effective patient involvement interventions in healthcare. This multi-faceted patient involvement intervention required a structured recruitment strategy, a full-day preparation meeting, consultation of a relatively large number of patients, small-group face-to-face deliberation between professionals and patients, financial compensation of participants, and moderation by an expert facilitator. Setting priorities with patients and professionals required more time and dedicated resources than with professionals alone. Existing patient involvement programs often lack some of these components, which may explain variations of observed effects in practice [21]. For example, it is common for one or two patient representatives to sit on advisory committees chaired by professionals, without structured patient preparation. Results of this trial points toward some ‘key ingredients’ that could support more effective patient involvement in collective healthcare improvement and policy decisions, and those factors have been described in details in a related publication [46].

### Study limitations and future research

The study sample size was small, and cluster randomization further reduces statistical power. However, the trial proved sufficient powered to test the impact of patient involvement on priority-setting, because the observed effect size was large. The small sample size may nonetheless have affected our ability to detect differences in professionals’ intention to use quality indicators.

While this study provides strong evidence that patient involvement can change improvement priorities, we cannot conclude that such differences will translate into changes in healthcare delivery. An important area for future research would therefore be to test, over a longer time period, whether patient involvement can transform healthcare services and have an impact on patient-oriented outcomes.

Also, while patient involvement had an important effect in this study, it is unknown if these results can be generalized to other contexts. This involvement intervention benefited from high-level policy support and was implemented within relatively small communities, which may have contributed to its effectiveness [47]. Caution is therefore required before extrapolating the effects observed in this study to other involvement programs and contexts.

## Conclusions

In conclusion, this study shows that patient involvement can change priorities driving healthcare improvement at the community level. Effective patient involvement requires time and dedicated resources. Future research should assess the generalizability of these findings to other contexts, and the impact of involvement interventions on patient care.

## Competing interests

All authors have completed the Unified Competing Interest form (available on request from the corresponding author; http://www.icmje.org/coi_disclosure.pdf) and declare no support or financial relationships with any organisations that might have an interest in the submitted work, and no other relationships or activities that could appear to have influenced the submitted work. The authors declare that they have no competing interests.

## Authors’ contributions

AB, PL, RL, JB, and RG designed the study, contributed to data collection and analysis, revised the final manuscript for important intellectual content, and approved its final version for publication. All authors had full access to the data. AB drafted the manuscript and is the guarantor. All authors read and approved the final manuscript.

## Supplementary Material

Additional file 1 Professionals' intention to use the selected quality indicators for healthcare improvement. Click here for file
