# Supplementary material for: Involving patients in setting priorities for healthcare improvement: a cluster randomized trial
Source: Implement Sci. 2014 Feb 20;9:24. doi: 10.1186/1748-5908-9-24 (PMC3936906; doi:10.1186/1748-5908-9-24)
Supplement: Additional file 1 — Professionals' intention to use the selected quality indicators for healthcare improvement. [file 1748-5908-9-24-S1.docx]

**Supplementary Appendix 1: Professionals' intention to use the selected quality indicators for healthcare improvement**

|  | | **Intervention** | **Control** | **Difference between intervention and control sites** | |
| --- | --- | --- | --- | --- | --- |
|  |  | Mean score (95% CI) | Mean score (95% CI) | Mean score differences (95% CI) | p value |
| **Professionals' perception of selected quality indicators as…** | |  |  |  |  |
| 1. | ...important improvement targets | 1.15 (0.98, 1.32) | 1.17 (0.96, 1.37) | 0.01 (-0.26, 0.28) | 0.92 |
| 2. | ...realist improvement targets | 1.42 (1.22, 1.62) | 1.56 (1.31, 1.8) | 0.13 (-0.18, 0.45) | 0.40 |
| 3. | …consensual improvement targets | 1.31 (1.09, 1.51) | 1.39 (1.14, 1.64) | 0.08 (-0.25, 0.41) | 0.62 |
| 4. | …credible improvement targets | 1.20 (0.10, 1.4) | 1.44 (1.2, 1.68) | 0.24 (-0.07, 0.56) | 0.12 |
| **Professionals' intention to use selected quality indicators…** | |  |  |  |  |
| 5. | ...to evaluate chronic care prevention and management | 1.48 (1.25, 1.70) | 1.56 (1.29, 1.82) | 0.08 (-0.27, 0.42) | 0.66 |
| 6. | ...to improve chronic care prevention and management | 1.36 (1.16, 1.56) | 1.39 (1.15, 1.62) | 0.03 (-0.28, 0.34) | 0.85 |
| 7. | ...to guide resource allocation | 1.92 (1.53, 2.3) | 1.94 (1.50, 2.39) | 0.03 (-0.56, 0.62) | 0.92 |
| 8. | ...to mobilize their organization around common goals | 1.36 (1.12, 1.59) | 1.28 (1.01, 1.55) | -0.08(-0.44, 0.27) | 0.64 |
| **Professionals' intention to report quality indicators' results to…** | |  |  |  |  |
| 9. | ...clinicians | 1.16 (0.99, 1.32) | 1.29 (1.09, 1.49) | 0.13 (-0.13, 0.40) | 0.31 |
| 10. | ...managers | 1.44 (1.15, 1.73) | 1.41 (1.06, 1.76) | -0.03 (-0.48, 0.42) | 0.90 |
| 11. | ...patients and the population | 1.44 (1.15, 1.72) | 1.72 (1.39, 2.05) | 0.28 (-0.15, 0.72) | 0.20 |

Legend: 1=Fully agree; 7=Fully disagree. Mean score differences > 0 favour intervention sites. All differences are above the statistical significance level of p<0,05.
